# In Silico Study of the Electrically Conductive and Electrochemical Properties of Hybrid Films Formed by Bilayer Graphene and Single-Wall Nanotubes under Axial Stretching

**DOI:** 10.3390/membranes11090658

**Published:** 2021-08-26

**Authors:** Michael M. Slepchenkov, Pavel V. Barkov, Olga E. Glukhova

**Affiliations:** 1 Institute of Physics, Saratov State University, 410012 Saratov, Russia; slepchenkovm@mail.ru (M.M.S.); barkovssu@mail.ru (P.V.B.); 2 Laboratory of Biomedical Nanotechnology, I.M. Sechenov First Moscow State Medical University, 119991 Moscow, Russia

**Keywords:** hybrid graphene/SWCNT films, axial stretching, electrical conductivity, quantum capacitance, density-functional tight-binding method, flexible electrodes

## Abstract

Using the self-consistent-charge density-functional tight-binding (SCC-DFTB) method, we studied the effect of axial stretching on the electrical conductivity and quantum capacitance of hybrid films formed by AB-stacked bilayer graphene and horizontally oriented single-walled carbon nanotubes (SWCNTs) with indices chirality (12, 6). The paper discusses several topological models of hybrid graphene/SWCNT (12, 6) films, which differ in the width of the graphene layer in the supercell and in the value of the shift between the graphene layers. It is shown that axial stretching has a different effect on the electrical conductivity and quantum capacity of the hybrid graphene/SWCNT (12, 6) film depending on the width of the graphene layer. For a topological model with a minimum width of the graphene layer (2 hexagons) under a 10% stretching strain, the transformation of bilayer graphene from planar to wave-like structures is characteristic. This transformation is accompanied by the appearance of the effect of anisotropy of electrical conductivity and a sharp decrease in the maximum of quantum capacitance. For a topological model with a graphene layer width of 4 hexagons, axial stretching, on the contrary, leads to a decrease in the effect of anisotropy of electrical conductivity and insignificant changes in the quantum capacitance. Based on the obtained results, the prospects for using hybrid graphene/SWCNT (12, 6) films as a material for creating flexible electrodes of supercapacitors are predicted.

## 1. Introduction

Currently, a rapidly developing field of modern electronics is flexible and stretchable electronics [1,2]. A key component in the design of many flexible electronics devices, including extremely highly relevant flexible batteries and supercapacitors, is the thin film electrode [3]. The choice of materials for the manufacture of thin-film electrodes plays an important role in achieving high-performance characteristics of flexible devices. The main requirements for materials in the manufacture of thin-film electrodes are high mechanical flexibility and extensibility and excellent electrical conductivity to ensure the required electrochemical characteristics as well as maintaining the structural integrity of the electrode during repeated deformations [3]. Traditional conductive materials for electrodes, such as polycrystalline films of metal oxides (indium tin oxide) or vaporized metals (Cu, Ag, or Au) and single-crystal inorganic semiconductors (silicon), are not suitable for making flexible electronic devices because of their rigidity and internal fragility [4]. Promising candidates for the fabrication of flexible electronics devices are carbon materials with many combined advantages, such as structural flexibility, good electrical conductivity, light weight, high chemical and thermal stability, ease of chemical functionalization, and the possibility of mass production [5,6]. Among carbon materials, graphene and carbon nanotubes (CNTs), which have high mechanical and electrically conductive properties, stand out first of all [7,8,9,10,11,12]. With the use of these materials, significant progress has been made in the development of flexible lithium-ion batteries and supercapacitors. Yoon et al. showed the efficiency of using CNT films heat-treated in a nitrogen atmosphere as an anode for flexible lithium-ion batteries [13]. The created electrode demonstrates an increase in the discharge capacity in the first cycle from 1300 to 3600 mAh g^–1^ in comparison with untreated CNT films due to the high crystalline perfection of the heat-treated films. In addition, heat-treated CNT-based film electrodes have twice the capacity than untreated electrodes (446 mAh g^−1^ at 0.5 C versus 165 mAh g^−1^). Sugiawati et al. found that pre-lithiated polymer-coated CNT anodes have a high capacity of 850 mAh g^−1^ at 1 C for 50 charge–discharge cycles [14]. CNT-based composites are no less promising anode materials for flexible batteries. One of such examples is a paper-like composite (NS@CNT) formed by the electrodeposition of nickel sulfide nanoparticles onto a thin CNT film. The flexible anode of a lithium-ion battery based on the NS@CNT composite shows a high specific capacity of ∼845 mAh g^−1^ at a current density of 60 mAg^−1^ [15]. Gilshteyn et al. reported the development of a high-performance tensile supercapacitor based on single-walled carbon nanotubes (SWCNT)-film electrodes and a boron-nitride nanotube separator [16]. The manufactured device retains 96% of its initial capacity after 20,000 electrochemical charge/discharge cycles with a specific capacity of 82 F g^−1^ and a low equivalent series resistance of 4.6 Ohm. Wang et al. presented a strong and flexible CNT film decorated with MnO_2_ nanosheets that exhibit superior Faraday pseudocapacitance when used as electrodes for flexible supercapacitors [17]. The proposed film shows a very high energy density of up to 27.14 W kg^−1^ at a power density of 571.3 W kg^−1^ and the ability to retain capacity after 8000 galvanostatic charge/discharge cycles, as well as excellent flexibility and long service life. 

Flexible graphene electrodes can also serve as an anode for flexible lithium-ion batteries [9,10,11,12]. When integrated with active materials, such as silicon, V_2_O_5_, MnO_2_, TiO_2_, and sulfur. to form a flexible electrode of batteries, graphene films demonstrate their unique properties, including excellent electrical conductivity, chemical stability, and high specific surface areas [18]. Wang et al. synthesized a flexible free-standing hybrid Fe_3_O_4_/graphene film for use as electrodes of lithium-ion batteries [19]. The fabricated electrode demonstrates a high specific capacity (1555 mAh g^−1^ at 100 mA g^−1^) and an excellent cyclic stability (940 and 660 mAh g^−1^ at 200 and 500 mA g^−1^ after 50 cycles, respectively). Qu et al. manufactured a supercapacitor electrode based on a graphene/g-C_3_N_4_ composite, which shows a specific capacity of 1500 mF cm^−2^, retaining 95% of the initial capacity after 5000 cycles and a maximum energy density of 0.075 mWh cm^−2^ [20]. Cao et al. have developed an electrode based on a flexible grapheme–ZnCo_2_O_4_ composite film that provides a high capacity of 874 mAh g^−1^ at a current density of 100 mA g^−1^ [21]. The main difficulty in using graphene as an electrode for lithium-ion batteries is associated with the agglomeration of graphene nanosheets, which prevents the transport of lithium ions [22]. One of the effective solutions to the formulated problem is the creation of hybrid layered structures, in which vertically or horizontally oriented CNTs are inserted between adjacent graphene monolayers. In these heterostructures, CNTs not only facilitate the penetration of the electrolyte, but also improve the conductivity and electrochemical characteristics of the electrodes. Specifically, Sun et al. fabricated a hybrid paper containing graphene nanosheets and CNTs that shows an increased capacity of 375 mAh g^−1^ at 100 mA g^−1^ [23]. Sun el al. used a graphene/CNT composite fiber as a supercapacitor electrode having a high specific capacitance of ∼31.50 F g^−1^ as compared to a supercapacitor based on CNT fibers (5.83 F g^−1^) [24]. A wide variety of options for constructing graphene–CNT architectures [25,26] opens up wide horizons for investigating the influence of the atomic structure features of a hybrid graphene–CNT composite on their electrophysical and electrochemical properties, including the aim of identifying ways to improve them. Computer modeling plays an important role in such studies. The thermal [27], electronic [28], and transport [29] of parallel and vertical graphene–CNT heterostructures are investigated by computer simulation methods. At the same time, there are very few in silico studies of the mechanical and electrochemical properties of graphene–CNT heterostructures [30,31,32]. Articles on this topic are mainly devoted to vertical heterostructures graphene–CNT composites (so-called pillared graphene). There are almost no such studies for parallel graphene–CNT heterostructures, although according to Zhang et al. [33], this topological type of hybrid graphene–CNT materials is the most widespread in practice. In this paper, in silico methods were used to study the effect of tensile deformation on the electroconductive and electrochemical properties of hybrid films formed by AB-stacked bilayer graphene and horizontally oriented SWCNTs (12, 6). 

## 2. Methods and Approaches

### 2.1. Calculation Details

The atomic structure of hybrid graphene/SWCNT films was carried out using the self-consistent-charge density-functional tight-binding (SCC-DFTB) method [34], implemented in the open access software package DFTB+ version 20.2 [35,36], with the Slater–Coster parameter set to be pbc-0-3 for all pair interactions [35]. The conjugate gradient method was used to optimize the structures. The geometrical parameters of the supercell structure were optimized, until the values of the forces between atoms were below 10^−5^ eV/Å. The SCC tolerance was set at 10^−5^ a.u. All calculations were performed at a temperature of 300 K. The SCC-DFTB method was chosen due to the polyatomic nature of supercells of the studied atomic configurations of hybrid graphene/SWCNT structures containing several hundred atoms, which increases the complexity of quantum calculations of such objects. Using the atomic structure of graphene as an example, it has been shown that the SCC-DFTB method is able to successfully reproduce structural and energy characteristics comparable in accuracy with the results of calculations by the density-functional tight-binding (DFT) method, but at lower computational costs [37]. 

The electrical conductivity was calculated within the Landauer–Buttiker formalism [38]. This formalism allows calculating the electron transmission function *T*(*E*) and static electrical conductivity *G*. The function *T*(*E*) is determined by the expression:

(1) $$T\left(E\right)=\frac{1}{N}\sum_{k=1}^{N}\mathrm{Tr}\left(\Gamma_{S}\left(E\right)G_{C}^{A}\left(E\right)\Gamma_{D}\left(E\right)G_{C}^{R}\left(E\right)\right),$$

where $G_{C}^{A}\left(E\right)$ and $G_{C}^{R}\left(E\right)$ are advanced and retarded Green matrices describing the contact with the electrodes; $\Gamma_{S}\left(E\right)$ and $\Gamma_{D}\left(E\right)$ are the levels broadening matrices for the source and the drain, respectively. Static conductivity is described by the expression:

(2) $$G=\frac{I}{V}=\frac{2e^{2}}{h}\int_{-\infty }^{\infty }T\left(E\right)F_{T}\left(E-E_{F}\right)dE,$$

where *E_F_* is the Fermi energy of the material of the contacts, *F_T_* is the thermal broadening function; *e* is the electron charge, *h* is the Planck’s constant, and *e*^2^/*h* is the quantum of conduction (this value is doubled to account for the electron spin).

In this study, the calculations of the transmission function *T*(*E*) were carried out for supercells of hybrid graphene/SWCNT films consisting of 250–300 atoms. In view of the large dimension of the considered electronic system, the original method for the accelerated calculation of the transmission function *T*(*E*) [39] was applied using the original Mizar program [40]. This method allows calculating *T*(*E*) for a small number of *k*-points of the first Brillouin zone and then interpolating it for any k-point of the first Brillouin zone and recovering the full transmission function *T*(*E*).

The calculation of the quantum capacity was carried out according to the following equation [41]:

(3) $$C_{Q}\left(V\right)=\frac{1}{mV}\int_{0}^{V}eD\left(E_{F}-eV\right)dV,$$

where *m* is the mass of the object, *V* is the displacement voltage, *e* is the elementary charge, *D* is the density of states (DOS) at the applied bias, and *E*_F_ is the Fermi level. 

### 2.2. Topological Models of Hybrid Graphene/SWCNT Films

**Figure 1:**
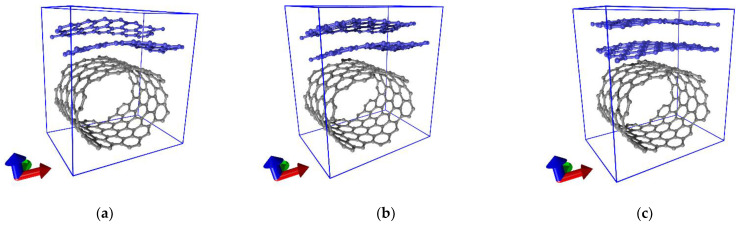
Topological models of supercells of hybrid films formed by bilayer graphene and single-walled carbon nanotubes (SWCNTs) (12, 6): (**a**) model V1; (**b**) model V2; (**c**) model V3. Hereinafter, the red axis corresponds to the X axis, the green axis corresponds to the Y axis, and the blue axis corresponds to the Z axis.

This study was carried out for three topological models of two-dimensional (2D) hybrid graphene/SWCNT films formed by AB-stacked bilayer graphene and SWCNTs (12, 6). The equilibrium configurations of the supercells of the hybrid graphene/SWCNT films are shown in Figure 1. The supercell of each of the models included fragments of bilayer graphene in the form of zigzag nanoribbons located above the surface of SWCNTs (12, 6) at a distance of 0.34 nm. The width of the graphene nanoribbon was 0.5 nm (2 hexagons) for the first model (model V1), 0.71 nm (3 hexagons) for the second model (model V2), and 0.92 nm (4 hexagons) for the third model (model V3). The graphene layers were displaced relative to each other in the direction of the armchair edge (along the Y axis), and the value of the shift *d* was different for each model: for the V1 model, *d* = 0.48 nm; for the V2 model, *d* = 0.27 nm; and for the V3 model, *d* = 0.06 nm. The distance between graphene layers along the Z axis (perpendicular to the nanotube axis) was 0.34 nm for all three topological models. Previously, these models were tested for energy stability and were used by us to study the electronic properties of hybrid graphene/SWCNT films [42]. The choice of SWCNTs (12, 6) and bilayer zigzag graphene nanoribbons is due to the following reasons. SWCNTs (12, 6) with a diameter of ~1.2 nm are among the most frequently synthesized SWCNTs with a high purity (over 90%) [43]. In addition, SWCNTs (12, 6) has a metallic type of conductivity, which indicates its prospects for use as electrodes in flexible electronics devices [44]. Since in many real experiments multilayer graphene is more stable than monolayer graphene, bilayer graphene is used in atomistic models of hybrid graphene–SWCNT films. The choice of graphene nanoribbons with zigzag edge is due to their unique transport properties, in particular, the presence of localized edge states with energies close to the Fermi level [45], which predetermines their broad prospects for use in electronic devices [46]. 

**Figure 2:**
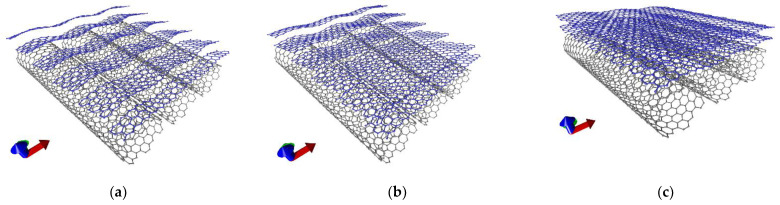
Topological models of extended fragments of hybrid films formed by bilayer graphene and SWCNTs (12, 6): (**a**) model V1; (**b**) model V2; (**c**) model V3.

Figure 1 shows that all three equilibrium configurations of the supercells of the graphene/SWCNT hybrid films were characterized by the deformation of the atomic structure of the SWCNTs (12, 6) and the bilayer graphene. This deformation was caused by the small length of graphene fragments and the large diameter of SWCNTs in the supercell and, as a consequence, by strong mutual influence nanotubes and graphene. Table 1 shows the data on the translation vectors *L*_x_ and *L*_y_ of the supercells of the hybrid graphene/SWCNT (12, 6) films. Topological models of hybrid graphene/SWCNT films obtained by translating supercells in two directions (X and Y) are shown in Figure 2.

## 3. Results and Discussion

### 3.1. Axial Stretching of Hybrid Graphene/SWCNT (12, 6) Films 

**Figure 3:**
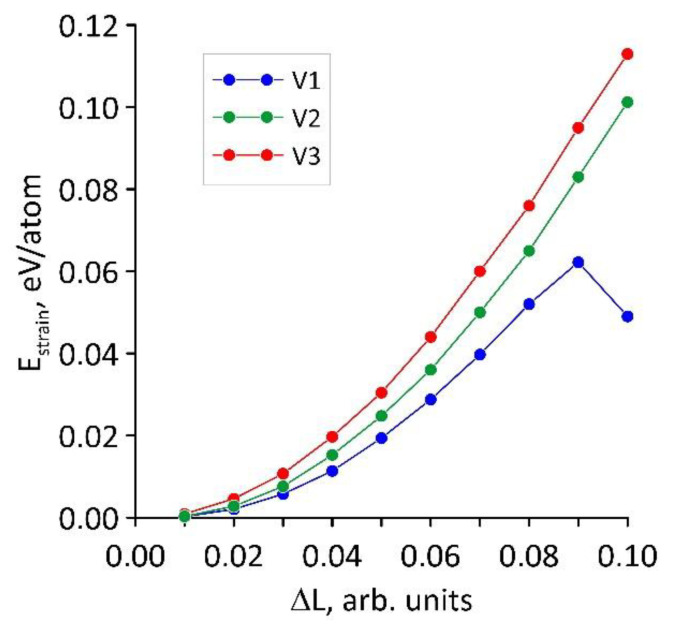
Graphs of the strain energy *E*_strain_ on the strain value Δ*L* in relative units for three topological models (V1, V2, V3) of hybrid graphene/SWCNT (12, 6) films.

As noted in the introduction, the main task of our study was to establish the effect of strain on the electrically conductive and electrochemical properties of hybrid films formed by bilayer graphene and SWCNTs (12, 6). We chose the stretching along the X axis, i.e., along the zigzag edge of bilayer graphene. Since the nanotube (12, 6) and bilayer graphene interacted by means of van der Waals forces in the supercell and the ribbon structure of graphene layers was taken into account, we chose such a variant of applying the straining force. The calculations were performed for the supercells of hybrid graphene/SWCNT films (12, 6) shown in Figure 1. During the simulation, the lengths of the translation vectors of the supercells were sequentially increased by 1% at each deformation step, after which the atomic structure was reoptimized in order to find a new energy-stable configuration. To prevent the atomic structure of the hybrid graphene/SWCNT (12, 6) film from returning to the initial state after bond stretching, the atoms at the edges of the supercell were rigidly fixed. The atoms being fixed were not included in the optimization. Each of the supercells of the topological models V1, V2, and V3 was stretched by 1–10% of its original size. The graphs of the strain energy *E*_strain_ on the strain value Δ*L* in relative units were constructed based on the results of the calculations. The strain energy was found from the difference between the total energy of the structure before and after axial stretching. Figure 3 shows the obtained dependences for models V1, V2, and V3. These graphs show that for models V2 and V3 in the considered range of stretching strain, an increase in the strain energy was observed according to a quadratic law, which corresponds to elastic strain. For model V1, when the structure was stretched up to 9%, a quadratic increase in the *E*_strain_ value was also observed; however, at a 10% stretching strain, a sharp decrease in the strain energy occurred. 

**Figure 4:**
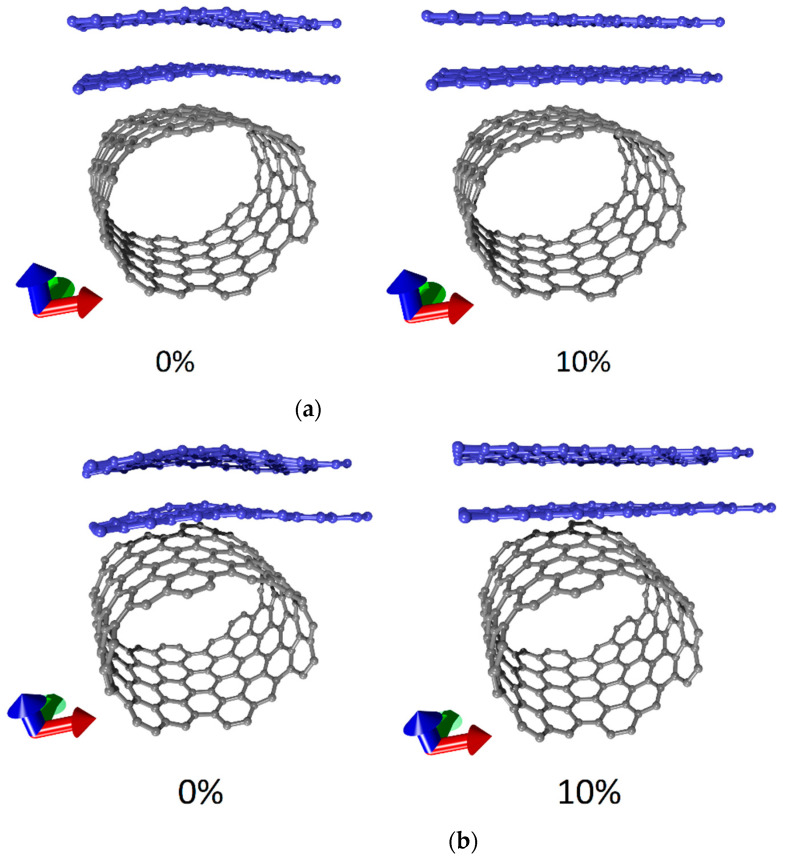

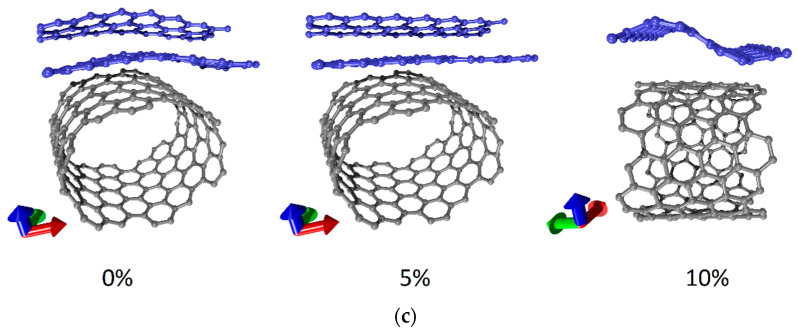
Changes in the atomic structure of supercells of hybrid films formed by bilayer graphene and SWCNTs (12, 6) upon axial stretching: (**a**) model V3; (**b**) model V2; (**c**) model V1.

To explain this behavior of the strain energy curves, let us trace the changes occurring in the atomic structure of supercells of models V1, V2, and V3 under stretching. Figure 4 shows the supercells of the hybrid graphene/SWCNT films (12, 6) in the absence of axial stretching and under stretching by a certain amount (10% for models V2 and V3; 5% and 10% for model V1). 

Figure 4 clearly demonstrates that the atomic structure of the supercells of models V3 (Figure 4a) and V2 (Figure 4b) changed under stretching in a similar way: initially, curvilinear graphene layers were straightened under the action of load. At a 10% stretching strain, this tendency can be traced especially clearly, so we present this case in the figure. For a long time, the atomic structure of the supercell of model V1 also demonstrated a tendency to straighten graphene layers upon stretching. In particular, the case of a 5% stretching strain for model V1 (see Figure 4c) was clearly similar to the cases of a 10% stretching strain for models V2 and V3. At a 10% stretching strain, noticeable changes occurred in the structure of the supercell of model V1: the planar configuration of graphene layers was replaced by a wavy one. As a result of the rearrangement of the atomic structure of the graphene layers, the strain energy of the supercell decreased and the strain ceased to be elastic. This result can be explained by the small width of the zigzag graphene nanoribbon in the supercell for the case of model V1. That is, the width of two hexagons was a characteristic topological parameter for the studied configurations of hybrid graphene/SWCNT (12, 6) films, at which the presence of strain caused a rearrangement of the atomic structure. It should be noted that the supercells of all three models retained their structural integrity over the entire range of stretching strain (1–10%). For models V1 and V2, the maximum C–C bond length did not exceed 0.15 nm; for model V3, it did not exceed 0.16 nm. At the same time, within the framework of this study, the task was not set to determine the values of deformations at which the bonds between carbon atoms are broken.

### 3.2. Effect of Axial Stretching on the Electrically Conductive and Electrochemical Properties of Hybrid Graphene/SWCNT (12, 6) Films

Let us proceed to study the effect of axial stretching on the electrically conductive and electrochemical properties of hybrid graphene/SWCNT (12, 6) films from the standpoint of assessing their potential prospects as a material for flexible electrodes. The electrically conductive properties were estimated by the value of the electrical resistance *R* for two directions of current transfer (*R*_x_ and *R*_y_): along the X axis (along the graphene layers) and along the Y axis (along the nanotube axis). We evaluated the electrochemical properties by the value of the quantum capacitance *C*_Q_. Table 2 summarizes the values of the resistances *R*_x_ and *R*_y_ for undeformed supercells and for selected cases of stretching strain (1%, 5%, and 10%), at which the most noticeable changes occurred in the atomic structure of the supercells of all three models.

After analyzing the data in the Table 2, the following conclusions can be drawn. For model V1, before the transformation of the planar configuration of graphene layers into a wavy one at a 10% stretching strain, the strain did not so noticeably affect the value of the resistance. In the direction of current transport along the Y axis, the resistance almost did not change, and in the direction of current transport along the X axis, it increased by ~0.9 kΩ at the initial moment of stretching. With the transformation of the planar configuration of graphene layers into a wavy one, a sharp jump in the resistance *R*_x_ was observed, which increased by a factor of 30. In this case, the value of the resistance *R*y increased only by ~3.5 kOhm. That is, for the case of the 10% stretching strain of model V1, the appearance of the effect of anisotropy of electrical conductivity and a significant deterioration of electrically conductive properties in the direction of current transfer along the X axis is characteristic. The resistance for model V2 for both the directions of current transfer turned out to be the least sensitive to stretching in the considered strain range. At a 10% stretching strain, the values of *R*_x_ and *R*_y_ decreased by only 0.2 kOhm in comparison with the resistance of the undeformed structure. For model V3, there was a clear improvement in the electrically conductive properties with increasing strain. If for an undeformed structure there was a pronounced anisotropy of electrical conductivity (*R*_x_ exceeded *R*_y_ by more than 10 times), then already at a 5% stretching strain *R*_x_ decreased by half, and at a 10% stretching strain it creased by about 4 times. The resistance *R*_y_ also decreased with increasing strain, and at a 10% stretching strain, it became half as much as in the *R*_y_ of the undeformed structure. The difference between the resistance values *R*_x_ and *R*_y_ was 5 times in the case of a 10% stretching strain, and it was ~8 times in the absence of deformation. It can be assumed that with the further stretching of the supercell of model V3, the tendency towards a decrease in the resistances *R*_x_ and *R*_y_ continue.

**Figure 5:**
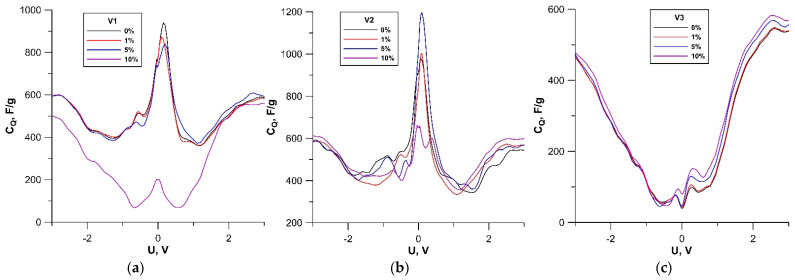
The graphs of the quantum capacitance *C*_Q_ of hybrid films formed by bilayer graphene and SWCNTs (12, 6) at different stretching strains: (**a**) model V1; (**b**) model V2; (**c**) model V3.

Let us now discuss how the quantum capacity *C*_Q_ of hybrid graphene/SWCNT (12, 6) films changed under axial stretching. Figure 5 shows the graphs of the quantum capacitance *C*_Q_ for models V1, V2, and V3 in the absence of axial strain and under stretching strain. As in the analysis of the behavior of electrical resistance, the cases of 1%, 5%, and 10% stretching strains were selected for demonstration. 

**Figure 6:**
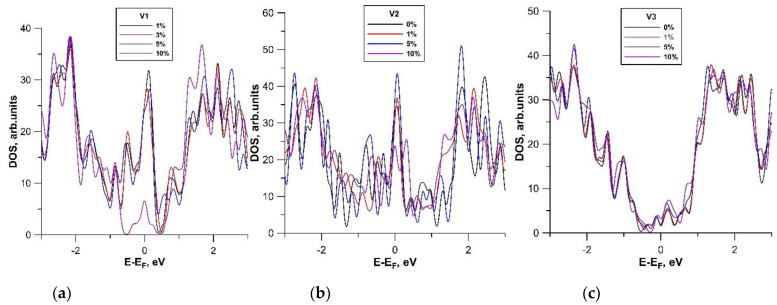
Density of states (DOS) of hybrid films formed by bilayer graphene and SWCNTs (12, 6) at different stretching strains: (**a**) model V1; (**b**) model V2; (**c**) model V3.

The graphs show that for models V1 (see Figure 5a) and V2 (see Figure 5b), the dependences of the quantum capacitance *C*_Q_ on the bias voltage were similar: the curve had a pronounced peak near 0 V. When stretched, the height of this peak began to change. At a 5% stretching strain, it decreased by 100 F g^−1^ for model V1 and it increased by 200 F g^−1^ for model V2. At a 10% stretching strain, a sharp decrease in the peak height was observed for both models: for model V1, its height decreased to 200 F g^−1^ and for model V2, to 650 F g^−1^. In the case of model V1, such a significant change in the peak height can be explained by the transformation of the planar configuration of graphene layers into a wavy one. In the case of model V2, this transition did not occur, but this value of stretching strain for the given topology of the hybrid graphene/SWCNT (12, 6) film was critical for the maximum value of the quantum capacitance. For model V3 (see Figure 5c), the quantum capacitance demonstrated stability under stretching over the entire strain range. With increasing stretching, only a slight increase in *C*_Q_ (by ~60 F g^−1^ at a 10% stretching strain) was observed near 0 V. Unlike models V1 and V2, the maximum value of the quantum capacitance *C*_Q_ for model V3 was reached not near 0 V, but at 2.5 V. At the same time, the maximum value of *C*_Q_ for model V3 (582 F g^−1^ at a 10% stretching strain) was noticeably lower as compared to the maxima of *C*_Q_ for models V1 (939 F g^−1^ in the absence of strain) and V2 (1197 F g^−1^ at a 5% stretching strain). To explain the revealed features of the behavior of quantum capacitance, we calculated the DOS distribution for models V1, V2, and V3 for the same cases of stretching strain as in Figure 5. The calculated DOS curves are shown in Figure 6.

Figure 6 demonstrates that the DOS profiles of each of the models repeated the corresponding quantum capacitance profiles shown in Figure 5, which is explained by the peculiarities of calculating *C*_Q_ according to Equation (3). For models V1 and V2, a high intensity of DOS peak was observed at the Fermi level (it shifted by 0 eV in the figure), which was also present in the graphs of *C*_Q_. As is known, the electronic states at the Fermi level make the decisive contribution to the conducting properties of a material. This explains the high values of the quantum capacitance at 0 V. For model V3, the DOS at the Fermi level was minimal, and the high-intensity DOS peaks were located far from the Fermi level. In this regard, the maximum of *C*_Q_ for model V3 was much lower than the maximum values of *C*_Q_ for models V1 and V2.

The reasons for the significant differences in the behavior of the electrically conductive and electrochemical properties between models V1 and V2 and model V3 lie in the peculiarities of their atomic structure. Let us clarify them using the example of Figure 2, illustrating extended fragments of hybrid graphene/SWCNT (12, 6) films obtained as a result of translations of supercells of models V1, V2, and V3. With a graphene nanoribbon width of 2 (model V1) and 3 hexagons (model V2) in the supercell, extended film fragments contained many rows of graphene nanoribbons oriented at an angle with respect to the SWCNT (12, 6) surface. With a graphene nanoribbon width of 4 hexagons (model V3), the extended fragments of hybrid films did not contain nanoribbons, but bilayer graphene sheets with linear dimensions that differed little from each other. That is, at this width, the translated fragments of graphene nanoribbons within each of the layers approached along the Y axis at a distance sufficient for the formation of covalent bonds with each other. Changing the graphene dimension led to such differences in the above properties between topological models. Thus, such topological features as the width of the graphene nanoribbon and the value of the shift between graphene layers are key factors in determining the electrically conductive and electrochemical properties of hybrid graphene/SWCNT (12, 6) films. 

## 4. Conclusions

Based on the SCC-DFTB calculation results, the regularities of changes in the atomic structure, as well as the electrically conductive and electrochemical characteristics of hybrid films formed by AB-stacked bilayer graphene and SWCNTs (12, 6) under axial stretching by 1–10%, were revealed. It was found that elastic strain was observed in almost the entire considered stretching range for the topological models under study. The exception was model V1 with the minimum width of the zigzag graphene nanoribbon (2 hexagons) in the supercell. For this model, the transformation of the planar configuration of bilayer graphene into a wavy one was revealed at a 10% stretching strain. The rearrangement of the atomic structure of bilayer graphene caused the appearance of the effect of the anisotropy of electrical conductivity and a fivefold decrease in the maximum value of the quantum capacity for this case of stretching strain. Model V2 with a graphene nanoribbon width of 3 hexagons in the supercell exhibited the best ability to retain high electrically conductive properties under stretching. The electrical resistance for this model in both the directions of current transfer changed by several tenths of 1 kOhm in the course of stretching. The same model was characterized by the maximum quantum capacity of 1197 F g^−1^ among all the considered models. This maximum was achieved when stretched by 5%. Model V3 with a graphene nanoribbon width of 3 hexagons in the supercell turned out to be the least sensitive to strain in the considered stretching range. In general, based on the obtained simulation results, a preliminary conclusion can be drawn about the possible prospects for the use of hybrid graphene/SWCNT (12, 6) films as a material for creating flexible electrodes for supercapacitors and batteries; however, it needs further experimental confirmation.

## Figures and Tables

**Table 1:** Geometric characteristics of supercells of hybrid films formed by bilayer graphene and SWCNTs (12, 6).

	Model V1	Model V2	Model V3
Number of atoms	252	280	308
*L_x_* (nm)	1.719	1.723	1.707
*L_y_* (nm)	1.135	1.134	1.110
*d* (nm)	0.48	0.27	0.06

**Table 2:** Electrical resistance values of hybrid graphene/SWCNT (12, 6) films under axial stretching.

	Model V1	Model V2	Model V3
0% stretching strain			
*R_x_* (kOhm)	7.53	6.22	125.89
*R_y_* (kOhm)	6.58	5.95	16.31
1% stretching strain			
*R_x_* (kOhm)	8.39	6.53	128.38
*R_y_* (kOhm)	6.50	5.90	15.87
5% stretching strain			
*R_x_* (kOhm)	7.68	6.00	64.19
*R_x_* (kOhm)	6.42	5.85	9.20
10% stretching strain			
*R_x_* (kOhm)	218.28	5.98	34.95
*R_y_* (kOhm)	10.154	5.75	7.179

## Data Availability

Not applicable.
